# Dialysis Adequacy and Risk of Dementia in Elderly Hemodialysis Patients

**DOI:** 10.3389/fmed.2021.769490

**Published:** 2021-11-30

**Authors:** Hyung Woo Kim, Jong Hyun Jhee, Young Su Joo, Ki Hwa Yang, Jin Ju Jung, Ji Hyeon Shin, Seung Hyeok Han, Tae-Hyun Yoo, Shin-Wook Kang, Jung Tak Park

**Affiliations:** ^1^Department of Internal Medicine, College of Medicine, Institute of Kidney Disease Research, Yonsei University, Seoul, South Korea; ^2^Division of Nephrology, Department of Internal Medicine, Gangnam Severance Hospital, Yonsei University College of Medicine, Seoul, South Korea; ^3^Division of Nephrology, Department of Internal Medicine, Yongin Severance Hospital, Yongin, South Korea; ^4^Healthcare Review and Assessment Committee, Health Insurance Review and Assessment Service, Wonju, South Korea; ^5^Quality Assessment Department, Health Insurance Review and Assessment Service, Wonju, South Korea; ^6^Quality Assessment Management Division, Health Insurance Review and Assessment Service, Wonju, South Korea; ^7^Department of Internal Medicine, College of Medicine, Severance Biomedical Science Institute, Brain Korea 21 PLUS, Yonsei University, Seoul, South Korea

**Keywords:** hemodialysis adequacy, dementia, hemodialysis, Alzheimer's disease, vascular dementia

## Abstract

**Objective:** Dementia is prevalent among elderly patients undergoing hemodialysis. However, the association between dialysis adequacy and the risk of dementia is uncertain.

**Methods:** A total of 10,567 patients aged >65 years undergoing maintenance hemodialysis who participated in a national hemodialysis quality assessment program were analyzed. The patients were classified into quartile groups based on single-pool Kt/V levels. The associations between single-pool Kt/V and the development of dementia, Alzheimer's disease (AD), and vascular dementia (VD) were examined.

**Results:** The mean age of the patients was 72.9 years, and 43.4% were female. The mean baseline single-pool Kt/V level was 1.6 ± 0.3. During a median follow-up of 45.6 (45.6–69.9) months, there were 27.6, 23.9, and 2.8 events/1,000 person-years of overall dementia, AD, and VD, respectively. The incidences of overall dementia, AD, and VD were lowest in the highest single-pool Kt/V quartile group. Compared with the lowest single-pool Kt/V quartile, the risks of incident overall dementia and AD were significantly lower in the highest quartile [sub-distribution hazard ratio (sHR): 0.69, 95% confidence interval (CI): 0.58–0.82 for overall dementia; sHR: 0.69, 95% CI: 0.57–0.84 for AD]. Inverse relationships were found between the risks of developing overall dementia and AD, and single-pool Kt/V. However, no significant relationship was observed between single-pool Kt/V levels and VD development.

**Conclusions:** Increased dialysis clearance was associated with a lower risk of developing dementia in elderly hemodialysis patients.

## Introduction

The number of elderly patients with end-stage kidney disease (ESKD) undergoing dialysis treatment is increasing worldwide (1, 2). Cognitive dysfunction, including dementia, is notably prevalent among elderly patients with ESKD (3–6). The 10-year risk of dementia after commencement of hemodialysis in patients aged 65 years is 20% and increases linearly with age (7). Cognitive dysfunction in patients with ESKD is closely related to adverse clinical outcomes, including functional impairment, frequent hospitalization, withdrawal from dialysis, and death, thus increasing the health care burden (8). Despite the high prevalence and clinical impact of cognitive dysfunction in patients with ESKD, the pathophysiology and related risk factors remain unclear.

The retention of molecular mediators in the circulation, which is potentially capable of brain damage, resulting from reduced kidney function has been proposed as one of the factors for kidney disease-related cognitive dysfunction (9, 10). Up to 9% of the uremic toxins accumulated in the circulation of kidney disease patients have been found to have neurological and central nervous system (CNS) effects (11, 12). Since the blood-brain barrier is less functional in advanced kidney disease patients, these toxins are capable of entering the CNS through diffusion and are suspected to have detrimental effects on the CNS, leading to cognitive dysfunction. Despite the plausible relationship between circulating uremic toxins and cognitive dysfunction in kidney disease patients, it is unknown whether increased clearance of these mediators through adequate dialysis affects cognitive function.

Therefore, in this study, the association between dialysis adequacy, measured by single-pool Kt/V (spKt/V), and the development of dementia was evaluated in elderly patients undergoing hemodialysis. This was done by assessing a national representative cohort of dialysis patients and claims data from a national health insurance database.

## Materials and Methods

### Data Source

More than 98% of the Korean population is enrolled in a mandatory National Health Insurance Service (NHIS) program, while the remaining citizens who are in the lowest-income bracket receive government medical aid benefits. The Health Insurance Review and Assessment Service (HIRA) is a national organization that reviews and evaluates healthcare costs and quality of care. The HIRA periodically performs a nationwide obligatory quality assessment for hemodialysis patients to ensure quality healthcare since 2010. All hemodialysis patients aged 18 years or older who undergo hemodialysis at least twice a week (eight times per month) as outpatients at a single healthcare provider during the quality assessment target period in Korea are entitled to the periodic hemodialysis quality assessment. The assessment collects information on demographics, cause of ESKD, dialysis vintage, and laboratory measurements, including hemoglobin, albumin, calcium, phosphorus, iron, ferritin and total iron binding capacity, blood pressure, erythropoietin use, intravenous iron use, and dialysis adequacy indices. This study used the 4th and 5th periodic hemodialysis quality assessment data from 2013 to 2015. Comorbidities and outcome diagnosis information were retrieved from the NHIS claims database. The study was conducted in accordance with the Declaration of Helsinki and approved by the Institutional Review Board of Yonsei University Health System (4-2019-0901). The requirement for informed consent was waived because of the retrospective nature of the study. De-identification was performed and data usage was permitted by the National Health Information Data Request Review Committee of the NHIS.

### Study Population

**Figure 1:**
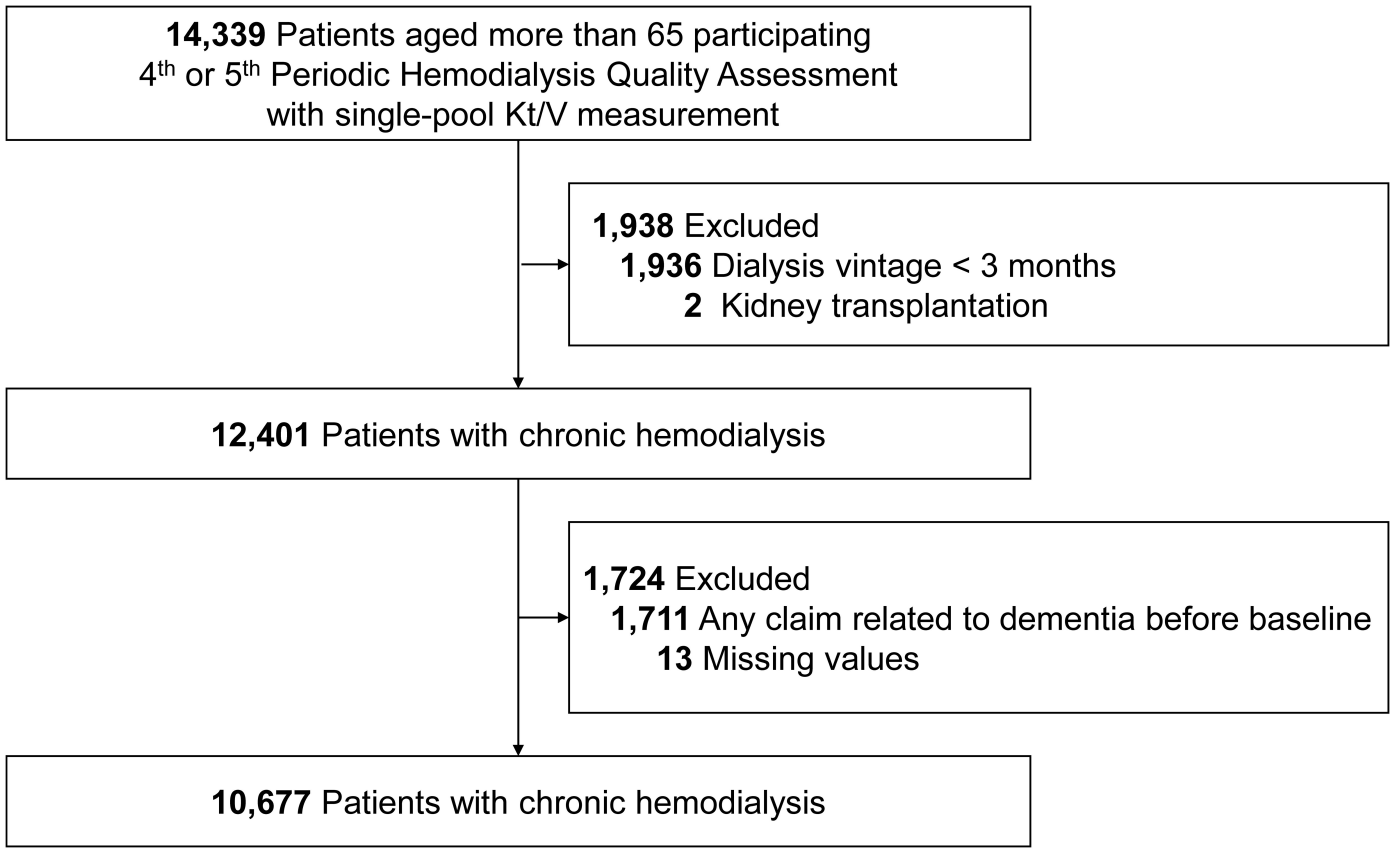
Study subjects.

Patients aged 65 years or older who were included in the 4th and 5th periodic hemodialysis quality assessment were initially screened. Patients with missing values of spKt/V, dialysis vintage <3 months, or a history of kidney transplantation were excluded. Patients who were diagnosed with dementia prior to the index date were also excluded. Furthermore, those who had dementia-related claims before the baseline were also excluded. A total of 10,677 patients on maintenance hemodialysis were included in the final analysis (Figure 1).

### Data Collection

The assessment dates of the 4th and 5th periodic hemodialysis quality assessment were considered as baseline. Economic status was classified based on the relative household income-derived health insurance premiums during the index year. Dialysis-related information included cause of ESRD, dialysis vintage, and type of vascular access [arteriovenous fistula, arteriovenous graft (AVG), or central catheter]. Comorbidities were defined as a record of at least one medical claim per year for a specific diagnosis. Diagnosis was defined based on the 10th revision of the International Statistical Classification of Diseases and Related Health Problems (ICD-10) codes (Supplementary Table 1). Blood samples were obtained immediately before a midweek dialysis session with overnight fasting. Evaluations were performed for hemoglobin; albumin; total calcium, and phosphorous; iron profile, including iron, ferritin, and total iron binding capacity.

### Assessment of Dialysis Adequacy

Dialysis adequacy values that were measured at baseline were used for the main analysis. The baseline spKt/V was assessed using the dialysis adequacy index. The spKt/V was calculated according to second generation logarithmic estimates of spKt/V; Kt/V = –ln (R – 0.008 × t) + (4 – 3.5 × R) × UF/W, where R is the ratio of pre- to post-hemodialysis concentrations of BUN, t is the dialysis session duration (in hours), UF is the amount of ultrafiltration (L) during the given hemodialysis session, and W is the post-hemodialysis weight (kg) (13). The urea reduction ratio (URR) was also assessed and used as a supporting dialysis adequacy index in the sensitivity analysis. The URR was calculated by dividing the difference in pre- and post-hemodialysis BUN concentrations by the pre-hemodialysis BUN concentration and multiplying by 100 (%) (14). Study subjects were classified into quartiles according to baseline spKt/V or URR levels.

### Outcome Measurement

The primary outcome was defined as the occurrence of dementia, which was defined as a claim record for ICD-10 dementia codes and a concurrent prescription record for dementia-related treatment. The dementia ICD-10 codes included F00 or G30 for Alzheimer's disease (AD), F01 for vascular dementia (VD), and F02, F03, or G31 for dementia not otherwise specified. Prescriptions for medications including donepezil, rivastigmine, galantamine, and memantine were considered to be dementia related. The study patients were followed up from baseline to the date of incident dementia or until the end of the study period (June 30, 2019).

### Statistical Analysis

Continuous variables were expressed as mean ± standard deviation, while categorical variables were expressed as absolute numbers with percentages. The Shapiro-Wilk test was used to determine the normality of the distribution of the parameters. Intergroup comparisons were performed using analysis of variance or Student's *t*-test for normally distributed continuous variables, while categorical variables were examined using the chi-square test or Fisher's exact test. Data that did not show a normal distribution were presented as medians with interquartile ranges and were compared using the Mann-Whitney *U*-test or Kruskal-Wallis test. To explore the association between dialysis adequacy and the development of dementia, sub-distribution hazard models were constructed. All-cause death was considered a competing risk (15). The sub-distribution hazard models were adjusted for age, sex, body mass index (BMI), pre-dialysis systolic blood pressure (SBP), economic status, cause of ESRD, dialysis vintage, vascular access type, Charlson Comorbidity Index (CCI; excluding diabetes and dementia), diabetes, hemoglobin, serum albumin, calcium, phosphorous, and use of intravenous iron or erythropoiesis-stimulating agent). To evaluate the relationship between the risk of incident dementia and spKt/V levels as a continuous variable, cubic spline analyses were conducted. A cubic spline was used, and each 1 percentile of the upper and lower spKt/V was eliminated to reduce distortion. Sensitivity analyses were performed to confirm these results. First, the association between spKt/V levels and the risk of dementia among subjects was assessed only for those who underwent hemodialysis at least three times per week. Second, the relationship between spKt/V levels and the risk of dementia was re-evaluated by censoring incident stroke history. Third, dialysis adequacy and the risk of dementia were further analyzed using the URR as a dialysis adequacy index. Fourth, evaluation considering kidney transplantation events as a competing risk was performed. Finally, analysis regarding spKt/V as a time varying variable was made to account for the effect of spKt/V change during follow-up in patients who underwent spKt/V measurements twice at a 2-year interval. All statistical analyses were performed using R (version 3.5.1; www.r-project.org; R Foundation for Statistical Computing, Vienna) and SAS Enterprise Guide, version 6.1 (SAS Institute). A *P* < 0.05 was considered as significant.

## Results

### Baseline Characteristics

The baseline characteristics of the study subjects according to the spKt/V level quartiles are shown in Table 1. The mean age was 72.8 ± 5.7 years and 6,057 (56.7%) were male. The mean spKt/V level was 1.6 ± 0.3. Patients in the higher spKt/V quartile were older and included more female patients and medical aid beneficiaries. In addition, more patients used AVG and had longer dialysis vintages in the higher spKt/V quartiles. The BMI, SBP, diastolic blood pressure, number of patients using antihypertensive agents, and CCI were lower in the higher spKt/V quartiles. Laboratory results revealed higher serum calcium levels and lower phosphorous levels in the higher spKt/V quartiles.

**Table 1:** Baseline characteristics according to quartile of spKt/V levels.

		**Quartile of spKt/V**				
**Characteristics**	**Total**	**Q1**	**Q2**	**Q3**	**Q4**	***P-*value**
	**(*N* = 10,677)**	**(*n* = 2,670)**	**(*n* = 2,669)**	**(*n* = 2,669)**	**(*n* = 2,669)**	
spKt/V	1.55 ± 0.31	1.23 ± 0.11	1.44 ± 0.05	1.61 ± 0.05	1.94 ± 0.32	<0.001
URR, %	72.23 ± 6.17	65.18 ± 4.19	70.34 ± 2.48	74.12 ± 2.57	79.47 ± 3.53	<0.001
Demographic data						
Age, years	72.8 ± 5.7	72.5 ± 5.6	72.5 ± 5.5	72.9 ± 5.7	73.3 ± 5.9	<0.001
Male	6,057 (56.7%)	2,206 (82.6%)	1,887 (70.7%)	1,329 (49.8%)	635 (23.8%)	<0.001
BMI, kg/m^2^	23.0 ± 3.2	23.9 ± 3.2	23.3 ± 3.1	22.9 ± 3.2	22.0 ± 2.9	<0.001
SBP, mmHg	138.9 ± 17.5	139.9 ± 17.2	139.2 ± 17.3	138.8 ± 17.3	137.7 ± 17.9	<0.001
DBP, mmHg	74.1 ± 11.5	75.4 ± 11.3	74.8 ± 11.2	73.5 ± 11.7	72.7 ± 11.5	<0.001
Medical aid beneficiaries	1,176 (11.0)	257 (9.6)	279 (10.5)	312 (11.7)	328 (12.3)	<0.001
Cause of ESKD						<0.001
Diabetic	5,217 (48.9)	1,463 (54.8)	1,388 (52.0)	1,286 (48.2)	1,080 (40.5)	
Hypertensive	3,126 (29.3)	697 (26.1)	750 (28.1)	809 (30.3)	870 (32.6)	
Glomerulonephritis	655 (6.1)	123 (4.6)	135 (5.1)	169 (6.3)	228 (8.5)	
Others	729 (6.8)	179 (6.7)	178 (6.7)	178 (6.7)	194 (7.3)	
Unknown	950 (8.9)	208 (7.8)	218 (8.2)	227 (8.5)	297 (11.1)	
Dialysis vintage, months	35.2 [16.5–74.0]	25.2 [13.0–55.0]	33.3 [16.2–70.5]	40.0[18.4–81.2]	45.1 [20.1–89.1]	<0.001
Vascular access type						<0.001
AVF	8,493 (79.5)	2,172 (81.3)	2,174 (81.5)	2,105 (78.9)	2,042 (76.5)	
AVG	1,987 (18.6)	439 (16.4)	454 (17.0)	518 (19.4)	576 (21.6)	
Central catheter	197 (1.8)	59 (2.2)	41 (1.5)	46 (1.7)	51 (1.9)	
Comorbidities						
CCI	5.1 ± 2.0	5.3 ± 2.0	5.2 ± 2.0	5.1 ± 2.0	4.9 ± 2.0	<0.001
Diabetes	5,675 (53.2)	1,576 (59.0)	1,523 (57.1)	1,393 (52.2)	1,183 (44.3)	<0.001
MI	600 (5.6)	160 (6.0)	163 (6.1)	143 (5.4)	134 (5.0)	0.256
CHF	2,791 (26.1)	717 (26.9)	700 (26.2)	689 (25.8)	685 (25.7)	0.759
CVD	1,646 (15.4)	434 (16.3)	410 (15.4)	393 (14.7)	409 (15.3)	0.486
PVD	3,139 (29.4)	830 (31.1)	771 (28.9)	800 (30.0)	738 (27.7)	0.039
Malignancy	1,057 (9.9)	277 (10.4)	284 (10.6)	271 (10.2)	225 (8.4)	0.030
Medication						
Antihypertensive agents	6,903 (64.7)	1,758 (65.8)	1,779 (66.7)	1,688 (63.2)	1,678 (62.9)	0.006
Statin	3,189 (29.9)	793 (29.7)	809 (30.3)	777 (29.1)	810 (30.3)	0.727
Aspirin	4,569 (42.8)	1,145 (42.9)	1,208 (45.3)	1,137 (42.6)	1,079 (40.4)	0.005
IV iron	1,249 (11.7)	305 (11.4)	309 (11.6)	319 (12.0)	316 (11.8)	0.929
ESA	9,266 (86.8)	2,302 (86.2)	2,295 (86.0)	2,318 (86.8)	2,351 (88.1)	0.103
Laboratory data						
Hemoglobin, g/dL	10.6 ± 1.0	10.6 ± 1.0	10.6 ± 1.0	10.6 ± 1.0	10.6 ± 1.0	0.029
Albumin, g/dL	3.9 ± 0.4	3.9 ± 0.4	3.9 ± 0.4	3.9 ± 0.4	3.9 ± 0.4	0.004
Calcium, mg/dL	8.8 ± 0.9	8.7 ± 0.9	8.8 ± 0.9	8.9 ± 0.9	8.9 ± 0.9	<0.001
Phosphorous, mg/dL	4.5 ± 1.4	4.6 ± 1.4	4.6 ± 1.4	4.5 ± 1.4	4.4 ± 1.4	<0.001
Serum iron	71.9 ± 32.0	72.0 ± 31.7	73.2 ± 32.1	72.3 ± 31.7	70.0 ± 32.5	0.014
TIBC	217.1 ± 44.0	221.6 ± 43.6	220.0 ± 44.0	215.1 ± 43.3	211.7 ± 44.2	<0.001
Ferritin	288.2 ± 286.1	256.0 ± 263.5	271.4 ± 269.6	297.2 ± 286.9	327.9 ± 316.5	<0.001

*Data are presented as mean (SD), median [IQR], or number (%)*.

*CCI was assessed without history of diabetes or dementia*.

*Medical aid was provided to the lowest income bracket*.

*spKt/V, single-pool Kt/V; URR, urea reduction ratio; BMI, body mass index; SBP, systolic blood pressure; DBP, diastolic blood pressure; ESKD, end-stage kidney disease; AVF, arteriovenous fistula; AVG arteriovenous graft; CCI, Charlson comorbidity index; MI, myocardial infarction; CHF, congestive heart failure; CVD, cerebrovascular disease; PVD, peripheral vascular disease; IV, intravenous injection; ESA, erythropoiesis stimulating agent; TIBC, total iron binding capacity*.

### Association Between Dialysis Adequacy and Dementia

During the median follow-up of 45.6 (45.6–69.9) months, there were 27.6, 23.9, and 2.8 events/1,000 person-years of overall dementia, AD, and VD, respectively. Among the groups stratified by spKt/V levels, the incidence of overall dementia and AD was lowest in the highest quartile. Compared with the lowest spKt/V quartile, the risks of developing overall dementia and AD were significantly lower in the highest quartile [subdistributed hazard ratio (sHR): 0.67, 95% confidence interval (CI): 0.57–0.80 for overall dementia; sHR: 0.68, 95% CI: 0.57–0.81 for AD]. However, no significant relationship was found with incident VD (sHR: 0.71, 95% CI: 0.44–1.16). This association remained significant even after making adjustments for confounding variables (sHR: 0.69, 95% CI: 0.58–0.82 for overall dementia; sHR: 0.69; 95% CI: 0.57–0.84 for AD). When spKt/V levels were treated as continuous variables, every increase of 0.1 in spKt/V was associated with a 3% risk reduction of incident overall dementia (sHR: 0.97, 95% CI: 0.95–0.99) and AD (sHR: 0.97, 95% CI: 0.94–0.99) after adjusting for confounding variables (Table 2). The hazard ratios of risk factors of dementia beside spKt/V are reported in Supplementary Table 2.

**Table 2:** Risk of dementia according to quartile of spKt/V.

	**spKt/V (per 0.1 increase)**	**Quartile of spKt/V**			
		**Q1**	**Q2**	**Q3**	**Q4**
Overall dementia					
Events	1,302	341	318	338	305
Event rates	27.6	28.3	26.9	29.0	26.3
Age, sex adjusted hazard ratio	0.97 (0.94–0.99)	1.00 (Reference)	0.88 (0.76–1.03)	0.85 (0.73–0.99)	0.67 (0.57–0.80)
Adjusted hazard ratio^a^	0.97 (0.95–0.99)	1.00 (Reference)	0.89 (0.77–1.04)	0.86 (0.73–1.01)	0.69 (0.58–0.82)
AD					
Events	1,137	285	291	298	263
Event rates	23.9	23.4	24.5	25.3	22.5
Age, sex adjusted hazard ratio	0.96 (0.94–0.99)	1.00 (Reference)	0.97 (0.82–1.14)	0.88 (0.74–1.04)	0.68 (0.57–0.81)
Adjusted hazard ratio^a^	0.97 (0.94–0.99)	1.00 (Reference)	0.98 (0.83–1.16)	0.90 (0.75–1.06)	0.69 (0.57–0.84)
VD					
Events	141	51	23	32	35
Event rates	2.8	4.0	1.8	2.6	2.8
Age, sex adjusted hazard ratio	0.99 (0.93–1.06)	1.00 (Reference)	0.46 (0.28–0.75)	0.64 (0.41–0.99)	0.71 (0.44–1.16)
Adjusted hazard ratio^a^	1.00 (0.94–1.06)	1.00 (Reference)	0.47 (0.28–0.77)	0.65 (0.41–1.03)	0.74 (0.44–1.26)

*Event rates are per 1,000 person-years*.

a *Adjusted for age, sex, BMI, pre-dialysis SBP, economic status, cause of ESKD, dialysis vintage, vascular access type, CCI (except for diabetes and dementia), diabetes, hemoglobin, serum albumin, calcium, phosphorous, and use of IV iron or ESA*.

*spKt/V, single-pool Kt/V;.AD, Alzheimer's disease; VD, vascular dementia; BMI, body mass index; SBP, systolic blood pressure; ESKD, end-stage kidney disease; CCI, Charlson comorbidity index; IV, intravenous injection; ESA, erythropoiesis stimulating agent*.

**Figure 2:**
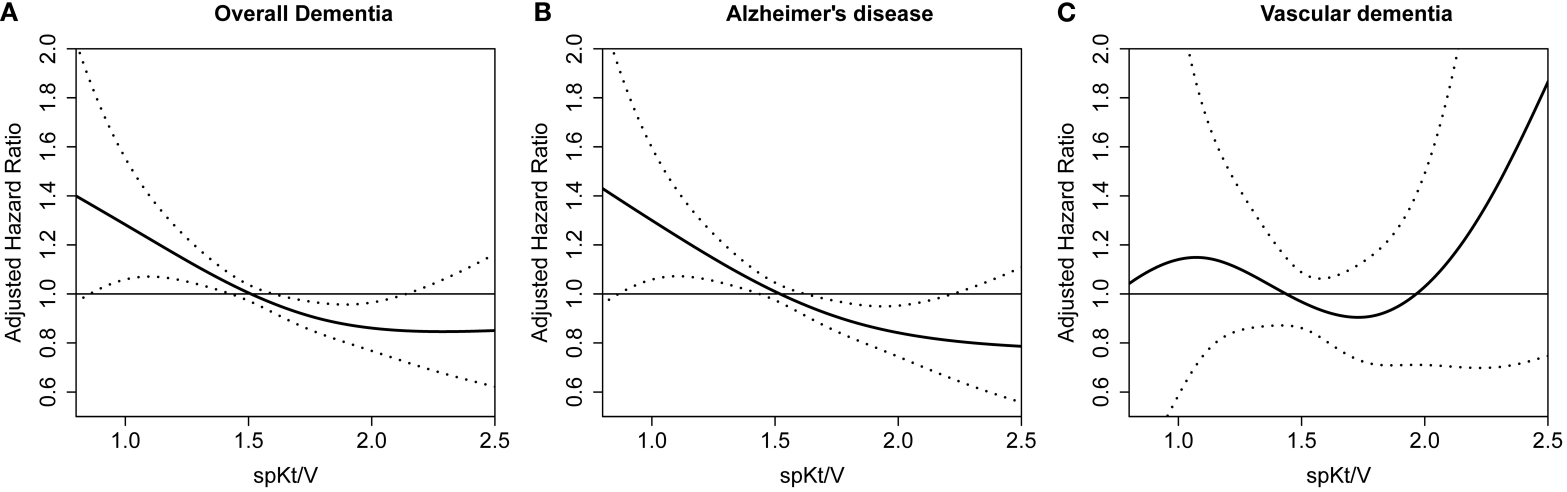
Restricted cubic spine plot for dementia occurrence according to spKt/V. **(A)** overall dementia **(B)** Alzheimer's disease **(C)** vascular dementia. spKt/V, single-pool Kt/V.

When the relationship between spKt/V and the adjusted hazard ratio for incident dementia was assessed using restricted cubic spline plots, the increase in spKt/V was associated with a linear decrease in overall dementia and AD risk. However, no clear relationship was found with VD (Figure 2).

### Subgroup Analysis

**Figure 3:**
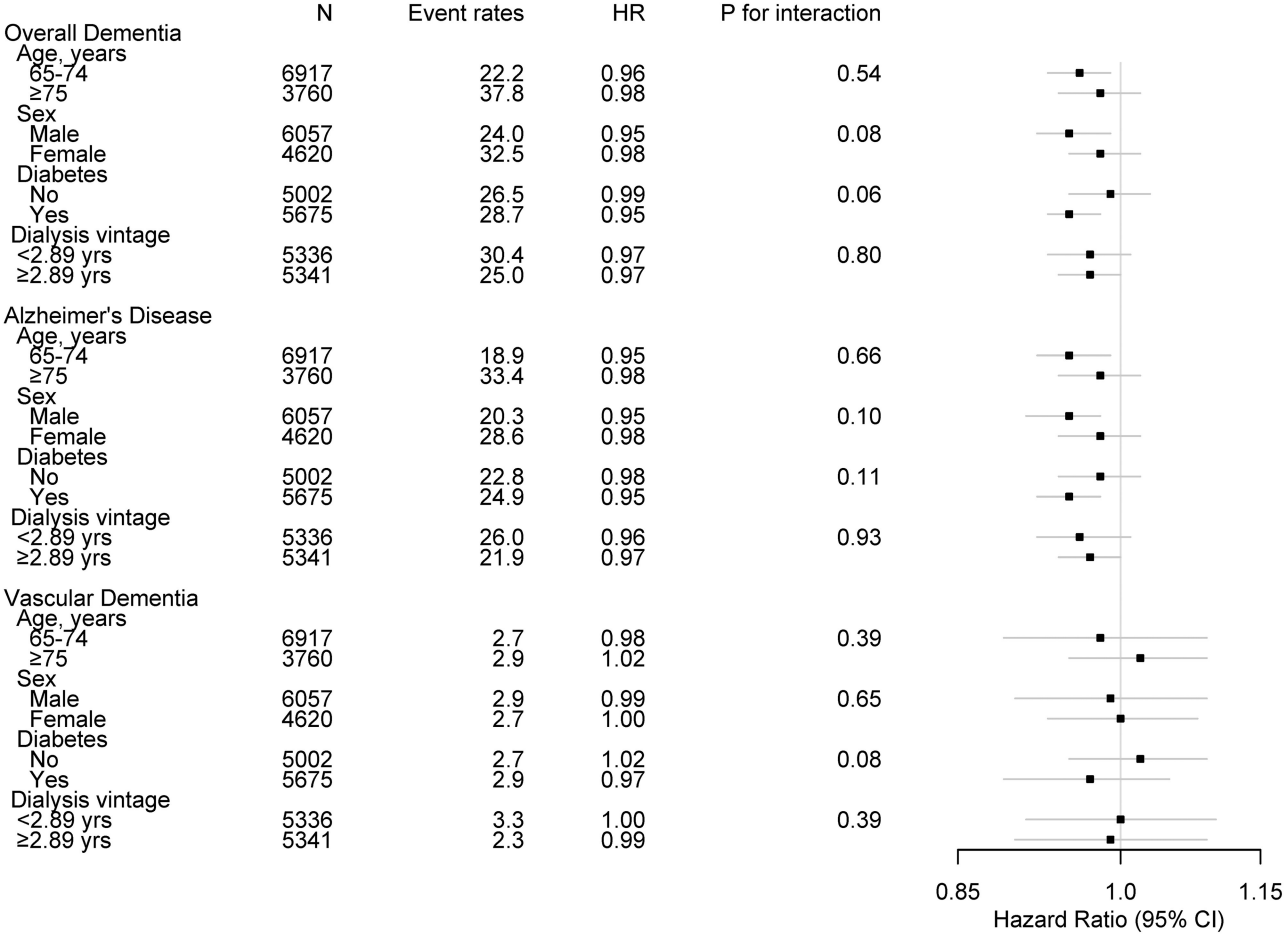
Subgroup associations of Kt/V with incident dementia. Event rates are per 1,000 person-years. HR was calculated with spKt/V levels treated as continuous variable for development of dementia. HR was adjusted for age, sex, BMI, pre-dialysis SBP, economic status, cause of ESKD, dialysis vintage, vascular access type, CCI (except for diabetes and dementia), diabetes, hemoglobin, serum albumin, calcium, phosphorous, and use of IV iron or ESA. HR, hazard ratio; spKt/V, single-pool Kt/V; BMI, body mass index; SBP, systolic blood pressure; ESKD, end-stage kidney disease; CCI, Charlson comorbidity index; IV, intravenous injection; ESA, erythropoiesis stimulating agent.

Subgroup analyses were performed in subjects stratified by age, sex, history of diabetes mellitus (no vs. yes), and dialysis vintage (median dialysis vintage of 2.89 years) (Figure 3). No significant interactions were found between the stratified variables and spKt/V for the incidence of overall dementia and AD, suggesting that the associations found in the main analysis are consistent regardless of age, sex, diabetes mellitus, and dialysis vintage.

### Sensitivity Analysis

When subjects who underwent dialysis at least three times per week were assessed, the risk of overall dementia (adjusted sHR: 0.71, 95% CI: 0.59–0.86) and AD (adjusted sHR: 0.74, 95% CI: 0.60–0.90) had significantly reduced in the highest spKt/V quartile compared to that in the lowest quartile. No significant association was found with VD risk (adjusted sHR: 0.65, 95% CI: 0.38–1.12). A significant relationship was noted when considering spKt/V as a continuous variable (per 0.1 increase in spKt/V, adjusted sHR: 0.97, 95% CI: 0.94–0.99 for overall dementia; HR: 0.96, 95% CI: 0.94–0.99 for AD) (Supplementary Table 3). Additionally, when incident stroke events were censored, the risk of overall dementia (adjusted sHR: 0.68, 95% CI: 0.57–0.82) and AD (adjusted sHR: 0.68, 95% CI: 0.56–0.83) was clearly reduced in the highest spKt/V quartile compared with the lowest quartile. No significant relationship was noted with VD risk (adjusted sHR: 0.74, 95% CI: 0.42–1.30). A significant relationship was noted with spKt/V when considered as a continuous variable (per 0.1 increase in spKt/V, adjusted sHR: 0.97, 95% CI: 0.95–0.99 for overall dementia; sHR: 0.97, 95% CI: 0.94–0.99 for AD) (Supplementary Table 4). Moreover, a similar finding to the main analysis was obtained when dialysis adequacy was assessed using the URR. The risk of overall dementia (adjusted sHR: 0.80, 95% CI: 0.87–0.96) and AD (adjusted sHR: 0.80, 95% CI: 0.66–0.98) was significantly lower in the highest URR quartile than in the lowest quartile. No clear relationship was found with VD risk (adjusted sHR: 0.89, 95% CI: 0.52–1.53). With URR considered as a continuous variable, a significant risk decrease was noted with an increase in URR (per 10-fold increase in URR, adjusted sHR: 0.86; 95% CI: 0.78–0.95 for overall dementia; HR: 0.85, 95% CI: 0.76–0.94 for AD) (Supplementary Table 5). When both all-cause death and kidney transplantation events were considered as competing risks, the risk of overall dementia and Alzheimer's disease were lower with increasing values of spKt/V (overall dementia: adjusted sHR, 0.97; 95% CI 0.95–0.99, Alzheimer's disease: adjusted sHR, 0.97; 95% CI, 0.94–0.99) (Supplementary Table 6). Furthermore, analysis of 2,892 patients who underwent spKt/V measurements twice at 2-year intervals showed that risk of overall dementia and Alzheimer's disease was lower with higher spKt/V values, when spKt/V was regarded as a time varying variable (overall dementia: adjusted sHR, 0.94; 95% CI, 0.90–0.99; Alzheimer's disease: adjusted sHR, 0.94; 95% CI, 0.89–0.99) (Supplementary Table 7).

## Discussion

In this study of a nationwide hemodialysis quality assessment database, a significant relationship between dialysis adequacy and the risk of developing dementia was revealed. The risk of incident overall dementia and AD was significantly lower in patients in the group with the highest spKt/V values compared than in that with the lowest spKt/V values. In addition, inverse relationships were found between the risk of developing overall dementia and AD and spKt/V. The significance of these relationships was maintained even after adjustments were made for confounding factors. Moreover, a similar association with the risk of incident dementia was also found when dialysis adequacy was assessed using the URR. However, a clear relationship between dialysis adequacy and the risk of developing VD was not observed.

Incident dementia was diagnosed in more than 12% of the study patients during a median follow-up duration of 45.6 months. The development of dementia has been found to be prevalent among elderly patients undergoing dialysis. In the general population, the prevalence of dementia has been reported to be ~5%; this increases with age, reaching 7.4% at the age of 70 years (16, 17). In comparison, in the Dialysis Outcomes and Practice Patterns Study, which evaluated a cohort of 16,694 patients on hemodialysis, the overall prevalence was increased to 20% in elderly patients (8). Similarly, in an evaluation of the United States Renal Data System, which included 356,668 dialysis patients aged 66 years or older, the lifetime risk of developing dementia was over 20% (7). In addition, in that study, the risk of mortality increased two-fold in those who were diagnosed with dementia, showing that the development of dementia also has a considerable impact on patient outcome. Kidney disease is one of the strongest risk factors for the development of cognitive impairment and dementia (3). In a longitudinal observational study in the general population, kidney disease was a more powerful risk factor for developing dementia than genetic factors and was only exceeded by stroke and chronic anxiolytic use (18).

The results of this study show that the risk of incident dementia is lower among patients with increased dialysis clearance, suggesting that zealous removal of uremic toxins may reduce the risk of developing dementia among elderly dialysis patients. This possibility is supported by the results of several previous studies. In the Dialysis Outcomes and Practice Patterns Study, residual renal function was a significant factor in lowering the risk of dementia, suggesting that uremic toxin excretion through the kidneys may play a role in the development of dementia in these patients (8). In addition, in a recent cross-sectional analysis of Chinese dialysis patients, low spKt/V was found to be related to cognitive impairment (19). Nonetheless, a previous cross-sectional analysis evaluating patients enrolled in the Frequent Hemodialysis Network trials failed to reveal a close association between dialysis clearance of urea and cognitive function (20). The discrepancy found in these studies could be explained by several aspects, including whether AD and VD were distinguished as causes of dementia in the analysis. However, the amount of clearance delivered would have played a major role. In this study, the risk of developing dementia decreased in patients whose mean spKt/V was 1.94 ± 0.32, which surpasses the minimal clearance amount recommended by current guidelines (21). The findings of this study suggest that although the current minimal dialysis clearance requirements are sufficient to reduce overall mortality (22), they could be insufficient to reduce the risk of dementia. However, further prospective intervention trials are required to confirm these findings.

The risk of AD, but not VD, decreased in patients with higher dialysis clearance. One of the causes of AD in dialysis patients has been attributed to the effects of circulating uremic toxins (10, 11). Circulating tumor necrosis factor has been found to impair neuronal synaptic function and memory at increased concentrations (23–25). In addition, neuropeptide Y (NPY), which is normally produced in peripheral nerve endings, accumulates in the circulation of chronic kidney disease and ESKD patients (26). High levels of NPY in the cerebrospinal fluid have been reported to be related to cognitive impairment in patients with subarachnoid hemorrhage (27). Other uremic toxins commonly accumulated in ESKD patients, such as asymmetric dimethyl arginine, hippuric acid, and indoxyl sulfate, have been found to be closely related to cognitive impairment (11, 28–30). However, VD is a common consequence of reduced cerebral blood flow (31). Factors contributing to acute and variable hemodynamic changes in dialysis patients, such as vascular access and large ultrafiltration volumes, could rather be more responsible for the aggravation of VD in these patients (32–34). Nonetheless, the effects of these hemodynamic change-promoting factors were not evaluated in detail in this study; hence, further investigations to assess these factors are needed.

This study has several limitations. First, due to the observational nature of the study, the cause-effect relationship cannot be determined. Further prospective studies modifying dialysis clearance are needed to confirm the influence on the development of dementia. Second, dementia was defined based on claim records. Therefore, the possibility of misclassification could not be ruled out. However, a previous study validating the accuracy of this working definition in the NHIS database revealed that the positive predictive value was 94.7%, suggesting that the chances of outcome misclassification would not be high (35). Lastly, changes in dialysis clearance during the follow-up period and residual renal function were not taken into account. Multiple potential factors that would affect dialysis clearance and residual renal function have been included as covariates to assure an independent association with clearance. However, it is indubitable that the consideration of these aspects would further clarify the findings of this study.

In conclusion, this large-scale cohort study of elderly hemodialysis patients demonstrated that increased dialysis clearance is associated with a lower risk of developing dementia. Ascertaining adequate dialysis clearance may lower the incidence of dementia and accordingly improve patient outcomes in elderly hemodialysis patients. Nonetheless, further investigations are needed to confirm these findings.

## Data Availability Statement

The datasets presented in this article are not readily available because the original data is available after getting approval from HIRA. The datasets are available online at: https://opendata.hira.or.kr.

## Ethics Statement

The studies involving human participants were reviewed and approved by Yonsei University College of Medicine. Written informed consent for participation was not required for this study in accordance with the national legislation and the institutional requirements.

## Author Contributions

HWK, JHJ, and JTP designed the study. JHJ wrote the manuscript. HWK analyzed the data. JTP reviewed and edited the manuscript. YSJ, KHY, JJJ, JHS, SHH, T-HY, and S-WK researched data and contributed to discussion. JTP took responsibility for the integrity of the data. All authors critically revised the manuscript for key intellectual content and approved the final version of the manuscript.

## Conflict of Interest

The authors declare that the research was conducted in the absence of any commercial or financial relationships that could be construed as a potential conflict of interest.

## Publisher's Note

All claims expressed in this article are solely those of the authors and do not necessarily represent those of their affiliated organizations, or those of the publisher, the editors and the reviewers. Any product that may be evaluated in this article, or claim that may be made by its manufacturer, is not guaranteed or endorsed by the publisher.
