# Supplementary material for: Dialysis Adequacy and Risk of Dementia in Elderly Hemodialysis Patients
Source: Front Med (Lausanne). 2021 Nov 30;8:769490. doi: 10.3389/fmed.2021.769490 (PMC8669136; doi:10.3389/fmed.2021.769490)
Supplement: Supplementary file 1 [file Data_Sheet_1.docx]

Supplementary Material

**Dialysis Adequacy and Risk of Dementia in Elderly Hemodialysis Patients**

Hyung Woo Kim, M.D.^1 †^, Jong Hyun Jhee, M.D., Ph.D.^2†^, Young Su Joo, M.D.^1,3^, Ki Hwa Yang, M.D., Ph.D.^4^, Jin Ju Jung, BSN^5^, Ji Hyeon Shin, BSN^6^, Seung Hyeok Han, M.D., Ph.D.^1^, Tae-Hyun Yoo, M.D., Ph.D.^1^, Shin-Wook Kang, M.D., Ph.D.^1,7^, and Jung Tak Park, M.D., Ph.D.^1*^

*^1^Department of Internal Medicine, College of Medicine, Institute of Kidney Disease Research, Yonsei University, Seoul, Korea*

*^2^Division of Nephrology, Department of Internal Medicine, Gangnam Severance Hospital, Yonsei University College of Medicine, Seoul, Korea*

*^3^Division of Nephrology, Department of Internal Medicine, Yongin Severance Hospital, Yongin, Gyeonggi-do, Republic of Korea*

*^4^Healthcare Review and Assessment Committee, Health Insurance Review and Assessment Service, Wonju, Korea*

*^5^Quality Assessment Department, Health Insurance Review and Assessment Service, Wonju, Korea*

*^6^Quality Assessment Management Division, Health Insurance Review and Assessment Service, Wonju, Korea*

*^7^Department of Internal Medicine, College of Medicine, Severance Biomedical Science Institute,*

*Brain Korea 21 PLUS, Yonsei University, Seoul, Korea*

**†These authors have contributed equally to this work.**

*** Correspondence:**Jung Tak Park, M.D., Ph.D.
[jtpark@yuhs.ac](mailto:jtpark@yuhs.ac)

**Supplementary Table S1.** International Classification of Diseases 10th Revision (ICD-10) for comorbidities

| **Diseases** | **ICD-10 codes** |
| --- | --- |
| Myocardial infarction | I21, I22, I252 |
| Congestive heart failure | I43, I50, I099, I110, I130, I132, I255, I420, I425, I426,  I427, I428, I429, P290 |
| Peripheral vascular disease | I70, I71, I731, I738, I739, I771, I790, I792, K551, K558,  K559, Z958, Z959 |
| Cerebrovascular disease | G45, G46, I60 ~ I69, H340 |
| Chronic Obstructive  Pulmonary Disease | J40 ~ J47, J60 ~ J67, I278, I279, J684, J701, J703 |
| Connective Tissue Disease | M05, M32 ~ M34, M06, M315, M351, M353, M360 |
| Peptic Ulcer Disease | K25 ~ K28 |
| Mild Liver Disease | B18, K73, K74, K700, K701 ~ K703, K709, K717, K713,  K714, K715, K760, K762 ~ K764, K768, K769, Z944 |
| Diabetes without complications | E100, E101, E106, E108 ~ E111, E116, E118 ~ E121, E126, E128 ~ E131, E136, E138 ~ E141, E146, E148,  E149 |
| Diabetes with complications | E102 ~ E105, E107, E112 ~ E115, E117, E122 ~ E125,  E127, E132 ~ E135, E137, E142 ~ E145, E147 |
| Paraplegia and Hemiplegia | G81, G82, G041, G114, G801, G802, G830 ~ G834,  G839 |
| Renal Disease | N18, N19, N052 ~ N057, N250, I120, I131, N032 ~  N037, Z490, Z491, Z492, Z940, Z992 |
| Cancer | C00 ~ C26, C30 ~ C34, C37 ~ C41, C43, C45 ~ C58,  C60 ~ C76, C81 ~ C85, C88, C90 ~ C97 |
| Moderate or Severe Liver  Disease | K704, K711, K721, K729, K765, K766, K767, I850, I859,  I864, I982 |
| Metastatic Carcinoma | C77 ~ C80 |
| Acquired immune deficiency  syndrome (AIDS) | B20 ~ B22, B24 |

**Supplementary Table S2.** HRs for dementia by patient characteristics

| **Variables** | **Adjusted sHR (95% CI)** |
| --- | --- |
| **spKt/V per 0.1 increase** | 0.97 (0.95-0.99) |
| **Age, per 1 year increase** | 1.05 (1.04-1.06) |
| **Female** | 1.45 (1.28-1.64) |
| **BMI, per 1 kg/m^2^ increase** | 0.99 (0.98-1.01) |
| **Pre-dialysis SBP, per 10 mmHg increase** | 0.99 (0.98-1.01) |
| **Medical Aids Beneficiaries** | 1.33 (1.14-1.56) |
| **Cause of ESKD** |  |
| Diabetes | 1.00 (reference) |
| Hypertension | 0.99 (0.80-1.22) |
| Glomerulonephritis | 1.02 (0.77-1.35) |
| Others | 0.87 (0.65-1.16) |
| Unknown | 0.95 (0.73-1.23) |
| **Dialysis Vintage, per 1 year increase** | 0.98 (0.97-0.99) |
| **Vascular access type** |  |
| AVF | 1.00 (reference) |
| AVG | 1.07 (0.94-1.23) |
| Central Catheter | 0.86 (0.58-1.27) |
| **CCI, per 1 increase** | 1.01 (0.98-1.05) |
| **Diabetes** | 1.08 (0.88-1.32) |
| **Hemoglobin, per 1 g/dL increase** | 1.01 (0.95-1.08) |
| **Serum albumin, per 1g/dL increase** | 0.95 (0.81-1.10) |
| **Serum calcium, per 1g/dL increase** | 1.05 (0.98-1.12) |
| **Serum phosphorus, per 1g/dL increase** | 1.00 (0.96-1.04) |
| **IV iron use** | 1.13 (0.95-1.36) |
| **ESA use** | 0.96 (0.80-1.15) |

***Note:*** All-cause death was treated as competing risk

***Abbreviations:*** spKt/V, single-pool Kt/V; HR, hazard ratio; CI, confidence interval; AD, Alzheimer’s disease; VD, vascular dementia; BMI, body mass index; SBP, systolic blood pressure; ESKD, end-stage kidney disease; CCI, Charlson comorbidity index; IV, intravenous injection; ESA, erythropoiesis stimulating agent

**Supplementary Table S3.** Risk of dementia according to quartile of spKt/V among subjects who underwent HD at least 3 times per week

| **spKt/V** | **Number of**  **events** | **Event rate** | **Model 1** | **Model 2** | **Model 3** |
| --- | --- | --- | --- | --- | --- |
|  |  |  | **HR (95% CI)** | **HR (95% CI)** | **HR (95% CI)** |
| Overall dementia |  |  |  |  |  |
| per 0.1 increase | 1157 | 27.3 | 0.96 (0.94-0.98) | 0.97 (0.94-0.99) | 0.97 (0.94-0.99) |
| Q1 | 303 | 28.1 | 1.00 (Reference) | 1.00 (Reference) | 1.00 (Reference) |
| Q2 | 284 | 26.7 | 0.90 (0.76-1.05) | 0.90 (0.76-1.06) | 0.90 (0.77-1.06) |
| Q3 | 292 | 27.8 | 0.83 (0.70-0.98) | 0.84 (0.70-0.99) | 0.84 (0.71-0.99) |
| Q4 | 278 | 26.7 | 0.70 (0.59-0.84) | 0.71 (0.59-0.86) | 0.71 (0.59-0.86) |
| AD |  |  |  |  |  |
| per 0.1 increase | 1006 | 23.5 | 0.96 (0.94-0.99) | 0.96 (0.94-0.99) | 0.96 (0.94-0.99) |
| Q1 | 249 | 22.7 | 1.00 (Reference) | 1.00 (Reference) | 1.00 (Reference) |
| Q2 | 260 | 24.3 | 1.00 (0.84-1.19) | 1.01 (0.84-1.20) | 1.01 (0.85-1.20) |
| Q3 | 257 | 24.2 | 0.88 (0.73-1.05) | 0.88 (0.74-1.06) | 0.89 (0.74-1.07) |
| Q4 | 240 | 22.8 | 0.72 (0.60-0.88) | 0.73 (0.60-0.90) | 0.74 (0.60-0.90) |
| VD |  |  |  |  |  |
| per 0.1 increase | 128 | 2.8 | 0.97 (0.90-1.03) | 0.97 (0.91-1.04) | 0.97 (0.91-1.04) |
| Q1 | 49 | 4.3 | 1.00 (Reference) | 1.00 (Reference) | 1.00 (Reference) |
| Q2 | 20 | 1.8 | 0.41 (0.24-0.69) | 0.42 (0.24-0.71) | 0.41 (0.24-0.70) |
| Q3 | 28 | 2.5 | 0.56 (0.36-0.89) | 0.58 (0.36-0.94) | 0.58 (0.36-0.94) |
| Q4 | 31 | 2.8 | 0.63 (0.38-1.03) | 0.66 (0.38-1.12) | 0.65 (0.38-1.12) |
| ***Note:*** Event rates are per 1000 person-years.  Model 1: Adjusted for age, and sex  Model 2: Adjusted for age, sex, BMI, pre-dialysis SBP, economic status, cause of ESKD, dialysis vintage, vascular access type, CCI (except for diabetes and  dementia), and diabetes  Model 3: Adjusted for Model 2 + hemoglobin, serum albumin, calcium, phosphorous, and use of IV iron or ESA  All-cause death was treated as competing risk  ***Abbreviations:*** spKt/V, single-pool Kt/V; HD, hemodialysis; HR, hazard ratio; CI, confidence interval; AD, Alzheimer’s disease; VD, vascular dementia; BMI, body mass index; SBP, systolic blood pressure; ESKD, end-stage kidney disease; CCI, Charlson comorbidity index; IV, intravenous injection; ESA, erythropoiesis stimulating agent | | | | | |

**Supplementary Table S4.** Risk of dementia according to quartile of spKt/V after censoring incident stroke

| **spKt/V** | **Number of**  **events** | **Event rate** | **Model 1** | **Model 2** | **Model 3** |
| --- | --- | --- | --- | --- | --- |
|  |  |  | **HR (95% CI)** | **HR (95% CI)** | **HR (95% CI)** |
| Overall dementia |  |  |  |  |  |
| per 0.1 increase | 1239 | 26.2 | 0.97 (0.94-0.99) | 0.97 (0.95-0.99) | 0.97 (0.95-0.99) |
| Q1 | 326 | 27.0 | 1.00 (Reference) | 1.00 (Reference) | 1.00 (Reference) |
| Q2 | 304 | 25.7 | 0.89 (0.76-1.04) | 0.89 (0.76-1.05) | 0.90 (0.77-1.05) |
| Q3 | 322 | 27.5 | 0.84 (0.72-0.99) | 0.85 (0.73-1.01) | 0.86 (0.73-1.01) |
| Q4 | 287 | 24.6 | 0.66 (0.56-0.79) | 0.68 (0.57-0.81) | 0.68 (0.57-0.82) |
| AD |  |  |  |  |  |
| per 0.1 increase | 1084 | 22.7 | 0.96 (0.94-0.99) | 0.97 (0.94-0.99) | 0.97 (0.94-0.99) |
| Q1 | 275 | 22.5 | 1.00 (Reference) | 1.00 (Reference) | 1.00 (Reference) |
| Q2 | 279 | 23.4 | 0.96 (0.82-1.14) | 0.97 (0.82-1.15) | 0.98 (0.82-1.16) |
| Q3 | 280 | 23.7 | 0.85 (0.72-1.01) | 0.87 (0.73-1.03) | 0.87 (0.73-1.04) |
| Q4 | 250 | 21.3 | 0.66 (0.55-0.80) | 0.68 (0.56-0.83) | 0.68 (0.56-0.83) |
| VD |  |  |  |  |  |
| per 0.1 increase | 131 | 2.6 | 0.99 (0.92-1.06) | 1.00 (0.93-1.06) | 1.00 (0.93-1.06) |
| Q1 | 47 | 3.7 | 1.00 (Reference) | 1.00 (Reference) | 1.00 (Reference) |
| Q2 | 21 | 1.7 | 0.45 (0.27-0.76) | 0.47 (0.28-0.79) | 0.47 (0.28-0.79) |
| Q3 | 32 | 2.6 | 0.69 (0.44-1.08) | 0.72 (0.46-1.15) | 0.72 (0.45-1.15) |
| Q4 | 31 | 2.5 | 0.67 (0.40-1.14) | 0.74 (0.42-1.30) | 0.74 (0.42-1.30) |
| ***Note:*** Event rates are per 1000 person-years.  Model 1: Adjusted for age, and sex  Model 2: Adjusted for age, sex, BMI, pre-dialysis SBP, economic status, cause of ESKD, dialysis vintage, vascular access type, CCI (except for diabetes and  dementia), and diabetes  Model 3: Adjusted for Model 2 + hemoglobin, serum albumin, calcium, phosphorous, and use of IV iron or ESA  All-cause death was treated as competing risk  ***Abbreviations:*** spKt/V, single-pool Kt/V; HR, hazard ratio; CI, confidence interval; AD, Alzheimer’s disease; VD, vascular dementia; BMI, body mass index; SBP, systolic blood pressure; ESKD, end-stage kidney disease; CCI, Charlson comorbidity index; IV, intravenous injection; ESA, erythropoiesis stimulating agent | | | | | |

**Supplementary Table S5.** Risk of dementia according to quartile of URR

| **URR** | **Number of**  **events** | **Event rate** | **Model 1** | **Model 2** | **Model 3** |
| --- | --- | --- | --- | --- | --- |
|  |  |  | **HR (95% CI)** | **HR (95% CI)** | **HR (95% CI)** |
| Overall dementia |  |  |  |  |  |
| per 10 increase | 1225 | 27.9 | 0.85 (0.78-0.94) | 0.86 (0.78-0.95) | 0.86 (0.78-0.95) |
| Q1 | 291 | 25.7 | 1.00 (Reference) | 1.00 (Reference) | 1.00 (Reference) |
| Q2 | 320 | 29.3 | 1.04 (0.89-1.22) | 1.05 (0.89-1.23) | 1.05 (0.89-1.23) |
| Q3 | 311 | 28.7 | 0.92 (0.78-1.08) | 0.93 (0.78-1.10) | 0.93 (0.78-1.10) |
| Q4 | 303 | 28.2 | 0.79 (0.66-0.94) | 0.80 (0.67-0.96) | 0.80 (0.87-0.96) |
| AD |  |  |  |  |  |
| per 10 increase | 1071 | 24.2 | 0.84 (0.76-0.93) | 0.85 (0.76-0.94) | 0.85 (0.76-0.94) |
| Q1 | 245 | 21.4 | 1.00 (Reference) | 1.00 (Reference) | 1.00 (Reference) |
| Q2 | 286 | 25.9 | 1.10 (0.93-1.31) | 1.11 (0.93-1.32) | 1.11 (0.93-1.32) |
| Q3 | 279 | 25.5 | 0.96 (0.81-1.15) | 0.98 (0.81-1.17) | 0.98 (0.82-1.17) |
| Q4 | 261 | 24.0 | 0.79 (0.65-0.95) | 0.80 (0.66-0.97) | 0.80 (0.66-0.98) |
| VD |  |  |  |  |  |
| per 10 increase |  | 2.8 | 0.99 (0.73-1.34) | 1.01 (0.73-1.40) | 1.01 (0.73-1.40) |
| Q1 | 39 | 3.2 | 1.00 (Reference) | 1.00 (Reference) | 1.00 (Reference) |
| Q2 | 31 | 2.6 | 0.81 (0.50-1.30) | 0.82 (0.51-1.32) | 0.81 (0.50-1.32) |
| Q3 | 27 | 2.3 | 0.70 (0.43-1.16) | 0.72 (0.43-1.22) | 0.72 (0.42-1.22) |
| Q4 | 33 | 2.9 | 0.86 (0.51-1.44) | 0.89 (0.52-1.53) | 0.89 (0.52-1.53) |
| ***Note:*** Event rates are per 1000 person-years.  Model 1: Adjusted for age, and sex  Model 2: Adjusted for age, sex, BMI, pre-dialysis SBP, economic status, cause of ESKD, dialysis vintage, vascular access type, CCI (except for diabetes and  dementia), and diabetes  Model 3: Adjusted for Model 2 + hemoglobin, serum albumin, calcium, phosphorous, and use of IV iron or ESA  All-cause death was treated as competing risk  ***Abbreviations:*** spKt/V, single-pool Kt/V; HR, hazard ratio; CI, confidence interval; AD, Alzheimer’s disease; VD, vascular dementia; BMI, body mass index; SBP, systolic blood pressure; ESKD, end-stage kidney disease; CCI, Charlson comorbidity index; IV, intravenous injection; ESA, erythropoiesis stimulating agent | | | | | |

**Supplementary Table S6.** Risk of dementia according to quartile of spKt/V. All-cause death and kidney transplantation were treated as competing risks.

|  | **spKt/V**  **(per 0.1 increase)** | **Quartile of spKt/V** | | | |
| --- | --- | --- | --- | --- | --- |
|  |  | **Q1** | **Q2** | **Q3** | **Q4** |
| Overall dementia |  |  |  |  |  |
| Age, sex adjusted hazard ratio | 0.97 (0.94-0.99) | 1.00 (Reference) | 0.88 (0.76-1.03) | 0.85 (0.73-0.99) | 0.67 (0.57-0.79) |
| Adjusted hazard ratio^a^ | 0.97 (0.95-0.99) | 1.00 (Reference) | 0.89 (0.76-1.04) | 0.86 (0.73-1.01) | 0.68 (0.57-0.82) |
| AD |  |  |  |  |  |
| Age, sex adjusted hazard ratio | 0.96 (0.94-0.99) | 1.00 (Reference) | 0.97 (0.82-1.14) | 0.88 (0.74-1.04) | 0.68 (0.56-0.81) |
| Adjusted hazard ratio^a^ | 0.97 (0.94-0.99) | 1.00 (Reference) | 0.98 (0.83-1.16) | 0.89 (0.75-1.06) | 0.69 (0.57-0.83) |
| VD |  |  |  |  |  |
| Age, sex adjusted hazard ratio | 0.99 (0.93-1.06) | 1.00 (Reference) | 0.44 (0.26-0.72) | 0.63 (0.41-0.98) | 0.70 (0.43-1.15) |
| Adjusted hazard ratio^a^ | 1.00 (0.94-1.06) | 1.00 (Reference) | 0.44 (0.27-0.74) | 0.65 (0.41-1.03) | 0.74 (0.43-1.24) |
| Death, n | 4599 | 1193 | 1161 | 1158 | 1097 |
| Kidney transplantation, n | 109 | 30 | 30 | 24 | 25 |
| ***Note:*** ^a^Adjusted for age, sex, BMI, pre-dialysis SBP, economic status, cause of ESKD, dialysis vintage, vascular access type, CCI (except for diabetes and dementia), diabetes, hemoglobin, serum albumin, calcium, phosphorous, and use of IV iron or ESA.  All-cause death and kidney transplantation were treated as competing risks  ***Abbreviations:*** spKt/V, single-pool Kt/V;.AD, Alzheimer’s disease; VD, vascular dementia; BMI, body mass index; SBP, systolic blood pressure; ESKD, end-stage kidney disease; CCI, Charlson comorbidity index; IV, intravenous injection; ESA, erythropoiesis stimulating agent | | | | | |

**Supplementary Table S7.** Risk of dementia among 2,892 patients underwent spKt/V measurements twice at two-year intervals.

| **spKt/V** | **Number of**  **events** | **Event rate** | **Age, sex adjusted** | **Full adjusted^a^** |
| --- | --- | --- | --- | --- |
|  |  |  | **sHR (95% CI)** | **sHR (95% CI)** |
| Overall dementia |  |  |  |  |
| per 0.1 increase | 351 | 22.4 | 0.95 (0.90-0.99) | 0.94 (0.90-0.99) |
| AD |  |  |  |  |
| per 0.1 increase | 305 | 19.3 | 0.95 (0.90-1.00) | 0.94 (0.89-0.99) |
| VD |  |  |  |  |
| per 0.1 increase | 40 | 2.4 | 0.95 (0.93-1.08)) | 0.97 (0.86-1.10) |
| ***Note:*** Event rates are per 1000 person-years. Age, spKt/V, BMI, pre-dialysis SBP, dialysis vintage, vascular access type, CCI (except for diabetes and dementia), diabetes, hemoglobin, serum albumin, calcium, phosphorous, and use of IV iron or ESA were treated as time-varying variables.  ^a^Adjusted for age, sex, BMI, pre-dialysis SBP, economic status, cause of ESKD, dialysis vintage, vascular access type, CCI (except for diabetes and dementia), diabetes, hemoglobin, serum albumin, calcium, phosphorous, and use of IV iron or ESA  ***Abbreviations:*** spKt/V, single-pool Kt/V; HD, hemodialysis; sHR, sub-distribution hazard ratio; CI, confidence interval; AD, Alzheimer’s disease; VD, vascular dementia; BMI, body mass index; SBP, systolic blood pressure; ESKD, end-stage kidney disease; CCI, Charlson comorbidity index; IV, intravenous injection; ESA, erythropoiesis stimulating agent | | | | |
